# Exogenous melatonin prolongs raspberry postharvest life quality by increasing some antioxidant and enzyme activity and phytochemical contents

**DOI:** 10.1038/s41598-024-62111-1

**Published:** 2024-05-20

**Authors:** Shirin Rahmanzadeh-Ishkeh, Habib Shirzad, Zahra Tofighi, Mohammad Fattahi, Youbert Ghosta

**Affiliations:** 1 Department of Horticultural Sciences, Faculty of Agriculture, Urmia University, Urmia, Iran; 2 Department of Pharmacognosy, Faculty of Pharmacy, Tehran University of Medical Sciences, Tehran, Iran; 3 Department of Plant Protection, Faculty of Agriculture, Urmia University, Urmia, Iran

**Keywords:** Rubus, Cold storage, N-acetyl-5-methoxytryptamine, PAL enzyme, Anthocyanin, Plant physiology, Secondary metabolism, Fungi

## Abstract

There is a growing trend towards enhancing the post-harvest shelf life and maintaining the nutritional quality of horticultural products using eco-friendly methods. Raspberries are valued for their diverse array of phenolic compounds, which are key contributors to their health-promoting properties. However, raspberries are prone to a relatively short post-harvest lifespan. The present study aimed to investigate the effect of exogenous melatonin (MEL; 0, 0.001, 0.01, and 0.1 mM) on decay control and shelf-life extension. The results demonstrated that MEL treatment significantly reduced the fruit decay rate (*P* ≤ 0.01). Based on the findings, MEL treatment significantly increased titratable acidity (TA), total phenolics content (TPC), total flavonoid content (TFC), and total anthocyanin content (TAC). Furthermore, the MEL-treated samples showed increased levels of rutin and quercetin content, as well as antioxidant activity as measured by 2,2-diphenyl-1-picrylhydrazyl (DPPH) and ferric reduction activity potential (FRAP). Additionally, the samples exhibited higher levels of phenylalanine ammonia-lyase (PAL) and catalase (CAT) enzymes compared to the control samples. Moreover, the levels of pH, total soluble solids (TSS), and IC_50_ were decreased in the MEL-treated samples (*P* ≤ 0.01). The highest amount of TA (0.619 g/100 ml juice), rutin (16.722 µg/ml juice) and quercetin (1.467 µg/ml juice), and PAL activity (225.696 nm/g FW/min) was observed at 0.001 mM treatment, while, the highest amount of TAC (227.235 mg Cy-g/100 ml juice) at a concentration of 0.01 mM and CAT (0.696 u/g FW) and TAL activities (9.553 nm/100 g FW) at a concentration of 0.1 mM were obtained. Considering the lack of significant differences in the effects of melatonin concentrations and the low dose of 0.001 mM, this concentration is recommended for further research. The hierarchical cluster analysis (HCA) and principal component analysis (PCA) divided the treatments into three groups based on their characteristics. Based on the Pearson correlation between TPC, TFC, TAC, and TAA, a positive correlation was observed with antioxidant (DPPH and FRAP) and enzyme (PAL and CAT) activities. The results of this study have identified melatonin as an eco-friendly compound that enhances the shelf life of raspberry fruits by improving phenolic compounds, as well as antioxidant and enzyme activities.

## Introduction

As the world’s population grows, food security will require further development in the agricultural portion^1^. In this regard, using elite and stable horticultural and agricultural cultivars can be helpful, but the control of limiting factors is undeniable. Pests and diseases are among the most important factors limiting the production and maintenance of crops^2,3^. Given the prevalent challenges in the agricultural sector, such as inadequate arable land, water scarcity, and soil salinity, addressing post-harvest issues has emerged as a prudent solution in the current era. Consequently, the reduction of post-harvest waste and the augmentation of farmers' incomes underscore the special significance of managing the storage of plant products^4–7^. In the post-harvest domain, concerns regarding food safety and environmental impact have prompted the utilization of edible coatings and plant growth regulators^8^. Plant growth regulators have long been known to play a significant role in extending post-harvest life^9^.

Melatonin (N-acetyl-5-methoxytryptamine: MEL) is a recently discovered plant growth regulator. This endogenous growth regulator, functioning as a phytohormone, has been recognized as a primary defense against oxidative stress, supported by other antioxidants. Its dual nature (hydrophilicity and lipophilicity) enables it to readily penetrate cell membranes and enter the cell^10^. Melatonin has been identified as a postharvest treatment that helps maintain the quality of crops, alleviates various abiotic stresses, reduces spoilage and decay caused by fruit pathogens, and increases the content of bioactive compounds. Several studies have shown that environmental stresses can elevate endogenous melatonin (MEL) levels. Additionally, the application of exogenous melatonin has been found to enhance resistance to both biotic (fungal) and abiotic (chilling) stresses,^11–14^ as well as to prolong the shelf life of various crops, including strawberry^15^, cucumber^16^, sweet cherry^17^, Chinese flowering cabbage^18^, ginger rhizomes^14^, and jujube^19^. This effect is attributed to the increased activity of the phenylalanine ammonia-lyase (PAL) enzyme, which plays a crucial role in the biosynthesis of phenols and flavonoids.

Small fruits, particularly raspberries, have garnered significant attention from consumers for their aromatic flavor and from researchers for their rich content of phytochemical compounds, including vitamin C, ellagic acid, anthocyanins, and phenols^20–24^. Fruits with high antioxidant content, such as raspberries, blackcurrants, blueberries, blackberries, and strawberries, are known to be rich in various phytochemicals^25^. Previous research has indicated that their antioxidant properties are at their peak when consumed fresh, underscoring the significance of storage and marketing for these fruits^26^. Various factors, including water loss, softening, mechanical injuries, and the presence of pathogens like *Botrytis cinerea* and *Rhizopus* sp., play a crucial role in determining the storage and marketing duration of raspberries^27,28^. However, advancements in biotechnology and physical techniques have made it possible to control these factors. One such technique involves the use of natural preservatives, including MEL, which is produced naturally by the plant itself.

In recent years, research efforts have primarily concentrated on the impact of MEL in increasing the post-harvest life of small fruits such as strawberries. However, there is a scarcity of studies examining the role of MEL in preserving phytochemicals and extending the shelf-life of raspberry fruits. Given the brief post-harvest lifespan of raspberries and the imperative to employ safe and health-conscious treatments that steer clear of the adverse effects associated with chemical preservatives, this study was undertaken to evaluate the influence of varying concentrations of MEL on cold storage, antioxidant, and enzyme activities, and phytochemical properties of raspberries.

## Materials and methods

### Plant materials

The Khan-Darasi region in Urmia, West Azerbaijan, Iran (Longitude: 45° 07′ 09’’, latitude: 37° 19′ 16’’, and 2392 m above sea level) is widely recognized as a significant natural habitat for raspberry growing in Iran. The *Rubus ulmifolius* subsp. *Sanctus* variety found in this region was identified by the herbarium of the Faculty of Pharmacy, Urmia University of Medical Sciences, Urmia, Iran, and a voucher herbarium sample has been registered under the number HUPS-359. In August 2021, uniform raspberry fruits, characterized by consistent size, color, and shape, were meticulously harvested from this locale. To minimize water loss and maintain fruit quality, the harvest was conducted in the early morning on a cloudy day and the freshly picked raspberries were quickly transferred in an icebox to a cold storage (4 ± 1 °C) at the Department of Horticultural Sciences of Urmia University.

### Melatonin treatment

**Figure 1:**
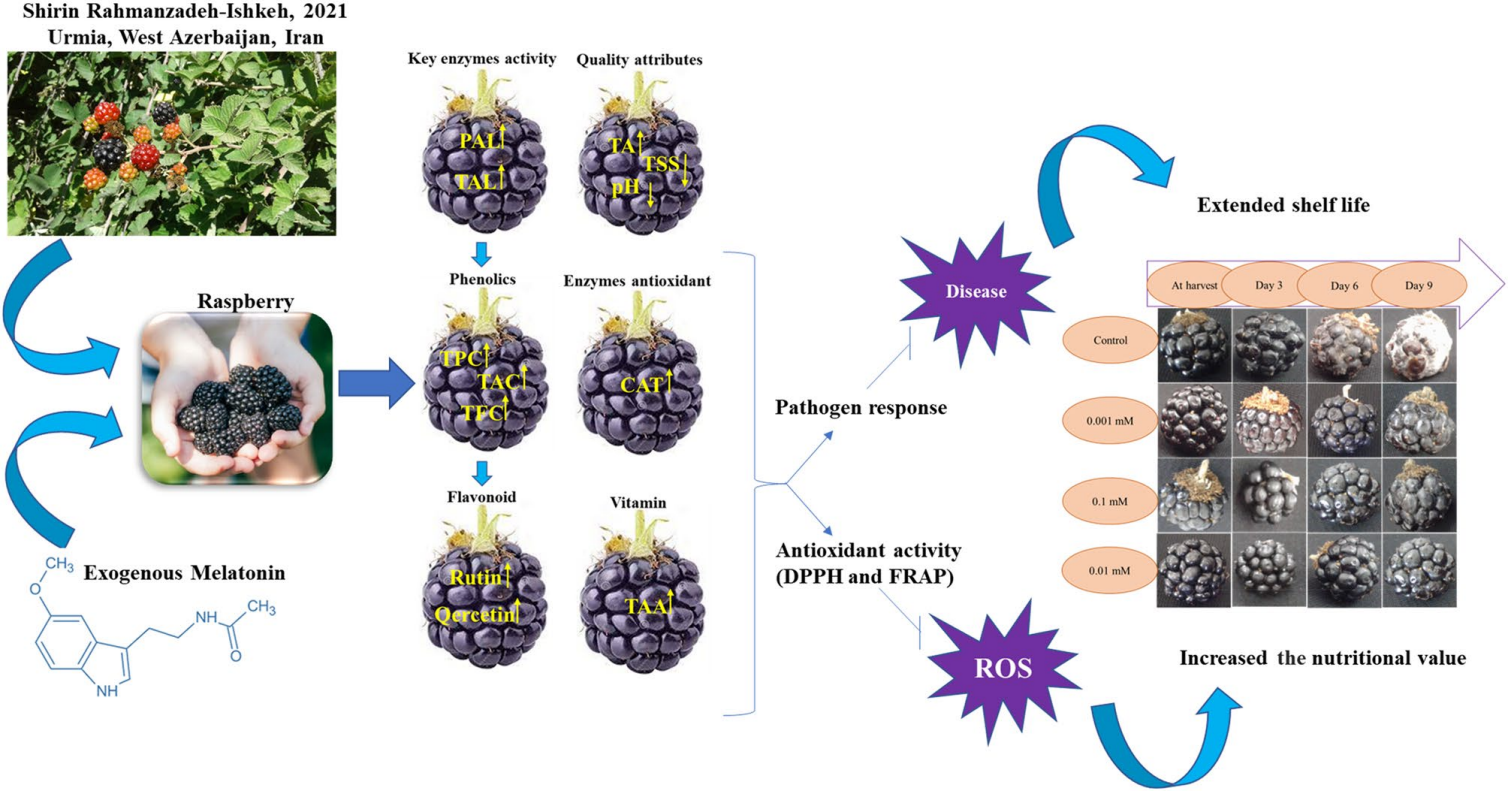
The different stages of the current study include the collection, treatment, and measurement of phytochemical properties and antioxidant and enzyme activities.

The MEL used in this experiment was purchased from Sigma-Aldrich Company. Different concentrations of MEL (0, 0.001, 0.01, and 0.1 mM) were prepared based on the molar mass. Subsequently, 60 g of raspberry fruits per experimental unit were immersed into the MEL solutions of each prepared concentration for five min. Following the treatment, the fruits were carefully transferred to sterilized containers and then placed in cold storage maintained at a temperature of 4 ± 1 °C, with relative humidity ranging between 90–95%. Each treatment was replicated four times, and the measured parameters were assessed at three-day intervals. The scheme of work is presented in Fig. 1.

### Chemical analyses (titratable acidity (TA), Total soluble solids (TSS), and pH)

For the chemical analyses, a portion of the fruits was manually squeezed according to standard procedure^29^, and the resulting juice was utilized to determine titratable acidity (TA), total soluble solids (TSS), and pH. Titratable acidity was assessed by diluting a 2 ml aliquot of raspberry juice in 20 ml of distilled water and titrating to a pH of 8.2 using 0.1 mol L^-1^ NaOH, with the results reported as g citric acid per 100 g fruit fresh weight. Furthermore, TSS was quantified using a refractometer (AZ-8601) at 20 °C and expressed as °Brix. The pH of the juice was measured using a pH meter (CG-824).

### Fungal decay measurement

The fungal decay of the fruits was visually assessed and quantified on days 3, 6, and 9 after storage using a scoring system ranging from 0 to 5. The fruits were classified into five categories based on the level of decay: 0–1 = No decay (excellent), 1–2 = Up to 5% decay (good), 2–3 = 5–20% decay (acceptable), 3–4 = 20–50% decay (bad), and 4–5 =  > 50% decay (unacceptable). A panel consisting of 10 individuals utilized the scoring index, and the average was calculated based on their evaluations.

### Phytochemical properties

#### Total phenolic content (TPC) measurement

To measure the total phenolic content, a modified version of the method described by Slinkard and Singleton^30^ was used. In this method, Folin Ciocalteu reagent was utilized. Initially, 10 µl of centrifuged fruit juice (11,000 g for 15 min at 4 °C) was combined with 180 µl of double distilled water. Subsequently, 1200 µl of Folin Ciocalteu (10%) was added to the mixture, followed by the addition of 960 µl of sodium carbonate (7.5%). The mixture was then kept in the dark for 10 min between each step, and the absorbance of the sample was read at 760 nm. Gallic acid was used as a standard, and the data were expressed as milligrams of gallic acid equivalent per ml of fruit juice (mg GAE/ml juice).

#### Total flavonoid content (TFC) measurement

The total flavonoid content was measured through a colorimetric assay at 380 nm, following the protocol outlined by Shin et al.^31^. Specifically, 30 µl of the fruit juice was mixed with 150 µl of 5% sodium nitrite, 300 µl of 10% aluminum chloride, and 1000 µl of sodium hydroxide (mol L^-1^). Following a 30-min incubation period in darkness, the sample volume was adjusted to 5 ml using double distilled water, and the absorbance was measured at 380 nm. The resulting data were then quantified based on quercetin and expressed as milligrams of quercetin equivalent per ml of fruit juice (mg QE/ml juice).

#### Total anthocyanin content (TAC) measurement

The pH differential method was used to measure the total ascorbic acid content (TAC). To achieve this, 100 μl of centrifuged fruit juice (centrifuged at 11,000 g for 15 min at 4 °C) was mixed with 2.5 ml of two buffers at pH 1 and 4.5. Then, the absorbance of the resulting samples was measured at 530 nm and 700 nm using a spectrophotometer. The final absorbance value was estimated using the following formula^32^:

A = [(A530 − A700) pH 1.0 − (A530 − A700) pH 4.5].

The total amount of anthocyanin was calculated using the formula: TAC content (mg L^-1^) = (A × MW × DF × 1000)/(*ε* × 1) based on mg equivalent of cyanidin 3-glucoside L^-1^ and then milligrams per 100 ml of fruit juice (mg Cy-g/100 ml juice), where,

A absorption, MW molecular weight of the cyanidin 3-glucoside, DF dilution factor, *ε* molar absorptivity.

#### Total ascorbic acid (TAA) measurement

Ascorbic acid content was measured using the 2,6-dichlorophenolindophenol (DCPIP) reagent according to the method of Bor et al.^33^. For extraction, one g of the fruit was homogenized with 30 ml of 1% (V/V) metaphosphoric acid using a mortar and pestle and then incubated in darkness for 30 min. The mixture was subsequently centrifuged at 11,000 g for 15 min at 4 °C. Then, 100 μl of the supernatant was combined with 2.5 ml of DCPIP, and the absorbance was read at 520 nm. The amount of AA was expressed as milligrams of ascorbic acid equivalent per g of fruit fresh weight (mg AA/g FW).

### Antioxidant capacity assay

#### Free radical scavenging activity (DPPH) and half-maximal inhibitory concentration (IC_50_)

The free radical scavenging activity of the fruit juice was measured using the DPPH method based on the principle introduced by Nakajima et al.^34^ with slight modification. For this means, 10 μl of the fruit juice was added to 2000 μl of DPPH solution (95% free radical: 6 × 10^–5^ mol L^-1^ in 80% methanol). The absorbance of the sample was measured at 517 nm after mixing the fruit juice with a DPPH solution and allowing it to react for a 30-min reaction period using a spectrophotometer (UV1901). In control treatments, the same procedure was performed using 80% methanol. The percentage of DPPH scavenging activity was then calculated using the formula provided:

$${\text{DPPHsc}}\% \, = \,\frac{{{\text{(Abs}}\,{\text{Control)t = x}}\,{\text{min - (Abs}}\,{\text{Sample)t = x}}\,{\text{min}}}}{{{\text{(Abs}}\,{\text{Control)t = x}}\,{\text{min}}}} \times {1}00\,$$

where Abs control = Control Absorption rate. Abs sample = Sample absorption rate.

Additionally, the half-maximal inhibitory concentration (IC50) of the fruit juices was calculated by measuring the antioxidant activity at five different concentrations (10, 20, 40, 60, and 80 µl).

### Ferric reducing antioxidant power assay

The ferric reducing antioxidant power (FRAP) assay was used to measure the antioxidant activity of the fruit juice^35^, 50 µl the fruit juice was diluted with 3 ml of the FRAP reagent [0.3 M acetate buffer (0.3 M, pH = 3.6), TPTZ: 2, 4, 6-tripyridyl-s-triazine (0.01 M in 0.04 M HCl) and FeCl_3_ 6H_2_O (0.02 M) (10:1:1, V/V/V)]. The resulting mixture was kept in a water bath at 37 ˚C for 30 min, and the absorbance was read at 593 nm. Iron sulfate was used as a standard, and the data were expressed as mmol Fe^++^/ml fruit juice.

### Enzymatic assay

#### Phenylalanine ammonia-lyase (PAL) and tyrosine ammonia-lyase (TAL) enzyme activity

The activities of phenylalanine ammonia-lyase (PAL: EC 4.3.1.24) and tyrosine ammonia-lyase (TAL: EC 4.3. 1.23) were determined following the method described by Beaudoin-Eagan and Thorpe^36^. For this purpose, 2 g of fruit tissue was homogenized in 4 ml of extraction buffer (0.05 M Tris–HCl (pH = 8.4)) containing 15 mM β-mercaptoethanol. The resulting supernatant from the centrifuged samples (12,000 rpm at 4 °C for 15 min) was employed to measure the activity of PAL and TAL enzymes. The assay buffer consisted of 6 μmol of L-phenylalanine to measure the PAL enzyme measurement, 5.5 μmol of L-tyrosine for TAL enzyme measurement, 500 μmol of Tris–HCl buffer (pH 8.01), and 100 μl of the enzyme extract. To calculate the activity of PAL (formation of *trans*-cinnamic acid) and TAL (formation of *p*-coumaric acid) enzymes, the prepared samples were incubated for 30 min at 37 °C. The reaction of both enzymes was stopped by adding 0.05 ml of 5 N HCl. The activities of PAL and TAL were read at 275 and 310 nm by a spectrophotometer (UV1901), respectively. Finally, the enzymatic activity for PAL and TAL was reported as nmol of *trans*-cinnamic acid g FW^-1^ min^-1^ and nmol of *p*-coumaric acid 100 g FW^-1^ min^-1^, respectively.

#### *Catalase* (*CAT*)* enzyme activity*

The enzyme activity of catalase (CAT: EC 1.11.1.6) was measured following the protocol outlined by Chance and Maehly^37^ using a colorimetric assay. Initially, one g of the raspberry fruit tissue homogenized with phosphate buffer (0.5 M phosphate buffer, pH 7.2 containing 1% (W/V) polyvinylpyrrolidone) was centrifuged at 10,000 g for 10 min at 4 °C to obtain the supernatant. Then, 0.1 ml of the enzyme extract was mixed with 2.5 ml sodium phosphate buffer (0.5 mM, pH = 7.0) and 0.3 ml H_2_O_2_ (15 mM), and the reduction in absorbance due to the decomposition of H_2_O_2_ was measured every 30 s. Finally, CAT activity was expressed in units per g FW^-1^.

#### Extraction and analysis of rutin and quercetin by HPLC/UV

To study the changes in rutin and quercetin compounds during cold storage, 0.5 g of the fruit tissue was homogenized in 5 ml of 80% methanol/water and formic acid (99–1, v/v). Ultrasonication (15 min at 30 ˚C) was utilized to enhance the extraction efficiency. The supernatant of the methanol extract of the samples was centrifuged (at 2000 g for 10 min at 4 °C) and filtered using a 0.45 µm filter.

For the separation, identification, and quantification of rutin and quercetin compounds, an HPLC device (HPLC–PDA, Water Alliance 2695 Separation Module) was employed, featuring a C18 (250 mm × 4.6 mm, 5 µm, Waters) column and was connected to a UV detector (Water 2487). A mobile phase of water with 0.02% trifluoroacetic acid (phases A) and methanol with 0.02% trifluoroacetic acid (phases B) was used, with a flow rate of 0.5 ml/min, an injection volume of 10 µl and a gradient of: (initial conditions: 20% B, 0–10 min; 20–30% B, 10–30 min; 30–60% B, 30–35; 60–100% B, 35–40 min; 100% B). All samples were injected in triplicate.

To establish a standard curve, rutin, and quercetin compounds were separately injected in six concentrations (5, 10, 20, 30, 40, and 60 mg/L). The standard calibration curve was obtained with a square correlation coefficient of *R*^2^ < 0.99.

#### Experimental design and statistical analyses

The present experiment was conducted using a completely randomized design, incorporating four concentrations of MEL and three storage durations, with each treatment replicated four times, each replication containing 60 g of raspberry fruits. Variance analysis and mean comparison using Duncan’s Multiple Range Test were conducted using SAS software (Version 9.1.3). Additionally, clustering, biplot, and correlation analyses were performed using R software (RStudio, version 1.2.5019, URL http://www.rstudio.com/). For the analysis of fungal decay, the Friedman test based on a randomized complete design was employed.

### Guideline statement

The authors confirm that the use of plants in the present study complies with international, national, and/or institutional guidelines.

## Results and discussion

### *Chemical analysis *(*TA, TSS, and pH*)

The pH and TSS levels in the control samples showed a significant increase during the cold storage period (*P* < 0.01), with the highest levels observed on the 9th for the control treatments. Treatment with melatonin at all concentrations (0.001, 0.01, and 0.1 mM) resulted in a deceleration of the rising trend of pH and TSS. This difference was statistically significant in all concentrations of MEL on the ninth day compared to the control (*P* < 0.01). However, no statistically significant difference was observed between different levels of MEL on the ninth day, except for the concentration of 0.001 mM in TSS.

**Figure 2:**
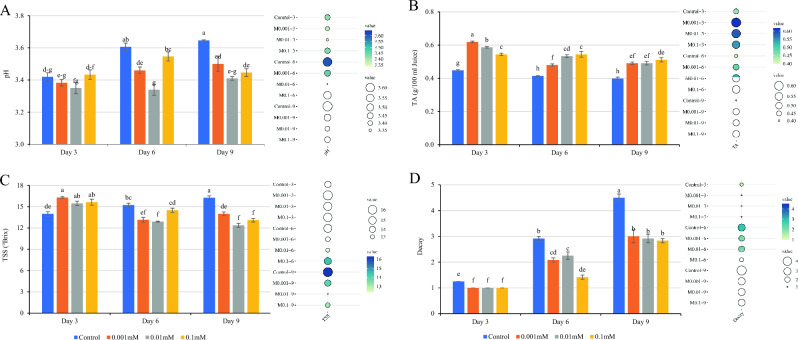
Effect of treatment with MEL on pH (**A**), titratable acidity (**B**), total soluble solids (**C**), and decay (**D**) of raspberry after cold storage. MEL: Melatonin. Columns followed by different letters in each group are significantly different at *P* < 0.01 according to Duncan’s multiple range test. Error bars represent standard errors for the selected chart series in each group (n = 4).

The objective of MEL treatment was to maintain the pH and TSS level at harvest until the 9th day of cold storage. On the 9th day, the lowest amount of pH (3.41) and TSS (12.367 ˚Brix) was detected in the 0.01 mM MEL treatment (Fig. 2A,C). Furthermore, MEL maintained helped in maintaining low pH and TSS levels, as well as the increasing trend of TA. Melatonin treatment increased the TA level compared to the control samples. In other words, the highest TA level (0.619 g/100 ml juice) was observed in the 0.001 mM MEL treatment on the 3rd day. However, this concentration of MEL could not maintain the TA level during the storage period, and on the ninth day, different concentrations of MEL were not statistically significantly.

The interrelation of TA, TSS, and pH levels in fruits is well-documented, with their changes being linked to senescence processes and fruit metabolism^38^. Previous studies have shown that the amount of TA in the strawberry fruit decreased during storage, and MEL treatment significantly reduced this decline^17^. By inhibiting respiration and preventing nutrient degradation, MEL has the potential to maintain TA, TSS, and pH levels during storage, thereby delaying senescence^17^. Similar findings have been reported in research on cucumber^16^, broccoli^39^, and peaches^40^ indicating an increase in pH, TSS, and TA with MEL treatment, However, another study demonstrated that MEL had no significant effect on the levels of the TSS and TA in ginger rhizome^14^.

### Fungal decay incidence

**Figure 3:**
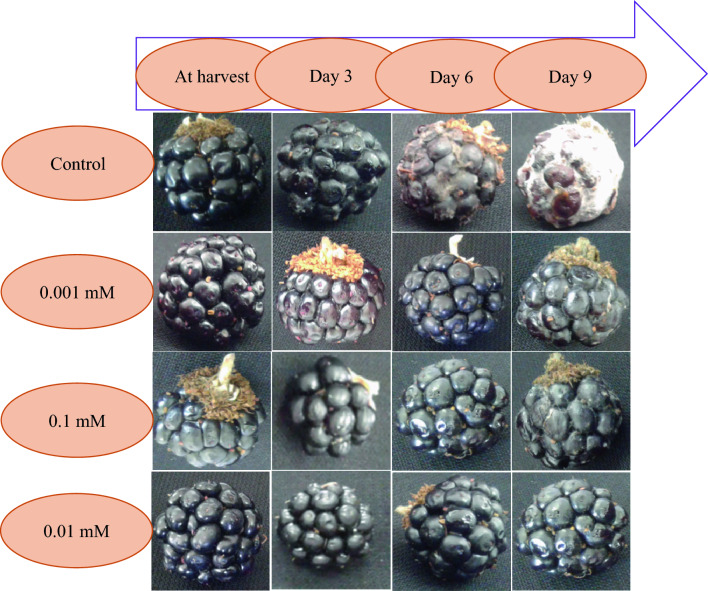
Effect of different MEL treatments on the fungal decay of raspberry fruit stored for 9 days at 4 ± 1 °C. Control refers to untreated raspberry. *MEL* Melatonin.

The small berry fruits industry, especially in the case of raspberry, faces challenges related to high water content and metabolic activity, which can increase sensitivity to fungal decay, including post-harvest fungi such as *Botrytis cinerea*, *Rhizopus* sp., *Penicillium expansum*, and *Aspergillus niger*^28,38^. In the present study, the highest amount of post-harvest decay was observed in the control treatment on the 9th day (4.5). However, the Findings of the present study revealed that MEL treatment could significantly reduce the rate of fungal decay in raspberry fruit compared to the control treatment (Fig. 3).

On the 9th day after storage, the application of MEL treatment (0.1 mM) reduced the decay rate by 37.04% compared to the control treatment, although no significant difference was observed between different concentrations of MEL (Fig. 2D). Previous research has consistently indicated that MEL can increase resistance to post-harvest diseases^13^.

The current study's findings align with previous reports on the effects of MEL treatment in reducing post-harvest decay in various fruits, including strawberries^41^, sweet cherries^17^, tomatoes^42^, ginger rhizome^14^, and apples^43^. Wang et al. ^17^ concluded that MEL treatment can preserve the integrity of cell membranes and prevent decay by modulating the respiration rate, maintaining endogenous MEL levels, enhancing defense-related enzymes and antioxidant activities, and reducing the accumulation of reactive oxygen species (O_2_^-^ and H_2_O_2_). Additionally, MEL treatment can stimulate the production of salicylic and jasmonic acids, induce the phenolic and phenylpropanoid synthesis pathways, and promote lignin production, thereby enhancing resistance against fungal diseases, especially those caused by *Botrytis cinerea*, *Fusarium* spp., and *Alternaria* spp., which are common post-harvest fungi of raspberries.

### Total phenolic and flavonoid contents

**Figure 4:**
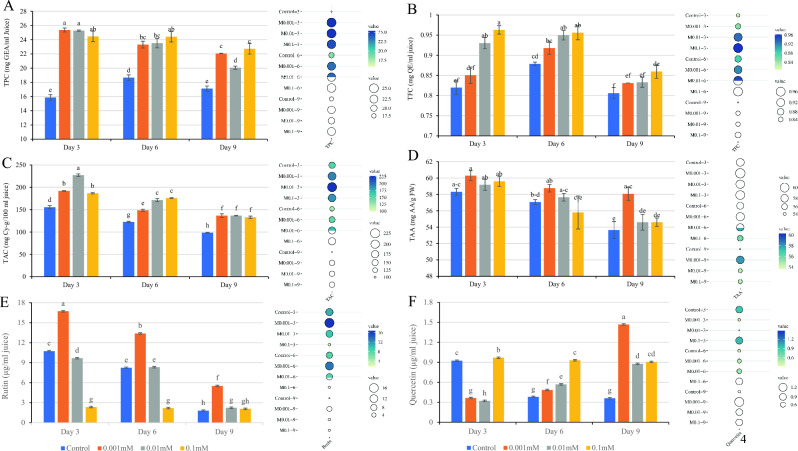
Effect of treatment with MEL on total phenolic content (**A**), total flavonoid content (**B**), total anthocyanin content (**C**), total ascorbic acid (**D**), rutin (**E**), and quercetin (**F**) of raspberry after cold storage. MEL: Melatonin. Columns followed by different letters in each group are significantly different at *P* < 0.01 based on Duncan’s multiple range test. Error bars demonstrate standard errors for the selected chart series in each group (n = 4).

As depicted in Fig. 4A,B, the application of MEL treatment resulted in a significant improvement in the total phenolic content (TPC) and total flavonoid content (TFC). The findings of the present study showed that, except for the concentration of 0.01 mM on the ninth day for TPC and the concentration of 0.001 mM on the third day for TFC, different concentrations of MEL did not have a significant effect on the TPC and TFC levels. However, across all days of storage, all concentrations of MEL led to a significant increase in the TPC and TFC levels compared to the control sample on the third, sixth, and ninth days. Notably, the 0.001 mM treatment on the third day for TFC did not result in a significant increase.

The nutritive value of stored horticulture crops typically diminishes over time due to metabolic activity and external stresses^44^. It is crucial to employ various treatments to slow down this process, and the use of safe preservatives has become increasingly important for food safety and health^45^. In the context of this study, MEL was utilized as a safe preservative, effectively maintaining the concentration of phenols and flavonoids in raspberry fruit during storage, thereby enhancing the nutritional quality of the fruits. While limited, existing reports on the effect of MEL on the preservation of fruit phenolic compounds during storage are promising. Previous studies showed that MEL treatment increased the concentration of phenols by increasing the activity of key enzymes such as Phenylalanine ammonia-lyase (PAL), Tyrosine ammonia-lyase (TAL), and Chalcone synthase (CHS) in the phenylalanine-propanoid pathway, this mechanism has been shown to effectively sustain the nutritional quality of fruits, as observed in litchi fruits^46^ and strawberries^41^ during storage, findings that align consistently with the results of the present study.

### Total anthocyanin and ascorbic acid content

The impact of MEL treatment on the amount of anthocyanin and ascorbic acid (AA) in raspberry fruit during storage is depicted in Fig. 4C,D. Notably, melatonin treatment effectively maintained the amount of anthocyanin during storage in comparison to the control treatments. The highest amount of anthocyanin (227.23 mg Cy-g/100 ml fruit juice) was observed on the third day in the 0.01 mM treatment. Furthermore, the results of the various concentrations of MEL treatments on the 9th day demonstrated preservation of more anthocyanin compared to the control treatment, and this difference was statistically significant (*P* < 0.05), However, no significant difference was observed between the different concentrations of MEL on the same day (Fig. 4C). It is important to note that anthocyanins are important components of raspberry fruits^47,48^, serving as potent antioxidants and contributing to the color of berries^23,49^.

In addition to their role in plant defense, anthocyanins also contribute significantly to the nutritional value of horticultural products^50^. Organic plants, which are grown without agricultural pesticides, tend to produce higher levels of anthocyanins as a natural defense mechanism due to the absence of chemical disease control^51^. However, the sensitivity of anthocyanins to various factors such as light, heat, and oxidative conditions means that they can degrade over time, impacting the overall nutritional composition of the produce^52^. In the present study, the MEL treatment's ability to maintain a high content of anthocyanins played a crucial role in controlling post-harvest decay and maintaining the nutritional value of the stored raspberry fruit. Furthermore, quantitative real-time polymerase chain reaction (qRT-PCR) studies have shown that MEL increases anthocyanin content by 69.4% through positive regulation of CsCHS and CsANS enzymes involved in anthocyanin biosynthesis^53^. These findings are in line with several other studies that have confirmed the positive effect of MEL on anthocyanin levels^53–56^. Melatonin treatment led to an increase in the level of ascorbic acid (AA) at the three time points studied (3, 6, and 9 d) compared to the control. However, this increase was not found to be statistically significant, except for the 0.001 mM treatment on the 9th day (Fig. 4D). Furthermore, the study results showed no statistically significant differences in the AA levels between the various concentrations of MEL on the third and sixth days. On the ninth day, the 0.001 mM treatment of MEL (yielding the highest amount of AA: 58.07 mg AA/g FW) showed a statistically significant difference compared to the concentrations of 0.01 and 0.1 mM as well as the control sample. Therefore, the 0.001 mM of MEL treatment had the most significant effect on maintaining AA levels over the ninth day. Ascorbic acid has been reported in various berries such as blackberry, raspberry, and gooseberry cultivars^21,57^. Some studies have also reported that the amount of AA in raspberries is higher than in fruits such as strawberries and oranges^58^ and kiwifruits^59^, which are traditionally known as the main sources of AA.

### Rutin and quercetin

The results of the study showed that the amount of rutin varied between 1.80 to 16.72 µg/ml juice (Fig. 4E). There was a decreasing trend in the amount of rutin in all treatments, with this trend increasing with higher MEL concentration and over time. The 0.001 mM MEL treatment on the 3rd day demonstrated the highest amount of rutin, showing the most significant impact on increasing and maintaining rutin levels over time. As depicted in Fig. 4F, the amount of quercetin in fruits treated with MEL exhibited an increasing trend over time, while in the control sample, this trend decreased. The highest amount of quercetin (1.46 µg/ml juice) was observed in the 0.001 mM MEL treatment on the 9th day. Rutin and quercetin are produced from the biosynthesis of flavonoids^60^. In this pathway quercetin is produced first, followed by rutin. The flavonol 3-O-glucosyltransferase enzyme converts quercetin to isoquercetin, and then the flavonol-3-O-glucoside L-rhamnosyltransferase enzyme converts it to rutin^61^. The results indicate that in the control samples, the levels of rutin and quercetin decreased during storage. However, with MEL treatment, the decreasing trend of rutin is accompanied by an increasing trend of quercetin over time. The results suggest that the MEL treatment may influence the enzymes involved in the production pathway of the rutin, shifting their effect towards quercetin. Additionally, it is hypothesized that quercetin transforms into rutin by adding sugar (glucoside), and the MEL treatment reduces the amount of rutin over time by reducing total soluble solids (TSS). These findings provide insights into the potential mechanisms by which MEL treatment influences the levels of rutin and quercetin in stored raspberry fruit.

### Antioxidant activity (2,2-diphenyl-1-picrylhydrazyl and ferric reduction activity potential) and half-maximal inhibitory concentration

**Figure 5:**
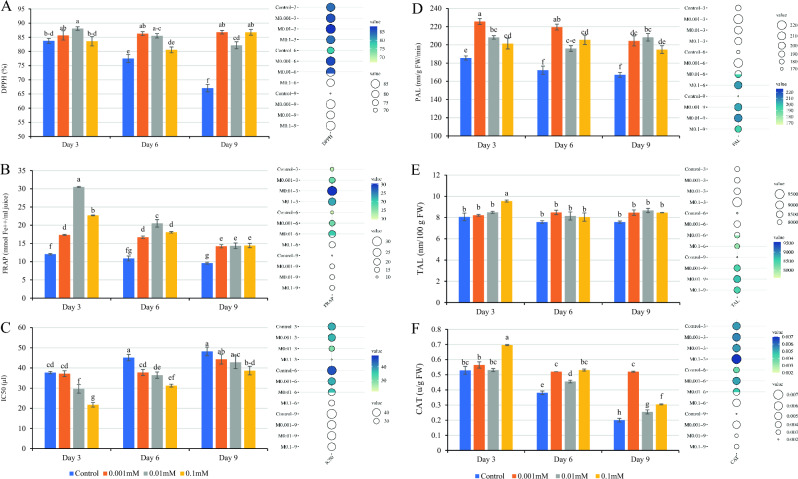
Effect of treatment with MEL on (**A**) antioxidant activity (DPPH), (B) antioxidant activity (FRAP), (**C**) half-maximal inhibitory concentration (IC_50_), (**D**) phenylalanine ammonia-lyase enzyme activity, (**E**) tyrosine ammonia-lyase enzyme activity, and (**F**) catalase enzyme activity of raspberry after cold storage. MEL: Melatonin; DPPH: 2-diphenyl-1-picrylhydrazyl; FRAP: Ferric reduction activity potential. Columns followed by different letters in each group are significantly different at *P* < 0.01 according to Duncan’s multiple range test. Error bars indicate standard errors for the selected chart series in each group (n = 4).

The study found significant differences in antioxidant activities and IC_50_ values based on the results. The effect of the MEL treatment on the amount of antioxidant activity by DPPH and FRAP methods were illustrated in Figs. 5A and 4B, respectively. The highest amount of antioxidant activity in the FRAP method was observed in the 0.01 mM treatment (FRAP: 30.50 mmol Fe^++^/ml juice) and in the same treatment in the DPPH method (DPPH: 88.09%) on the 3rd day. This was without a statistically significant difference with 0.001 mM treatment. The lowest amount was obtained in the control sample on the 9th day (DPPH: 67.07% and FRAP: 9.60 mmol Fe^++^/ml juice). Based on the data presented in Fig. 5C, the amount of IC_50_ demonstrated an increasing trend over time, with the increasing trend being steeper in the control samples.

In summary, the study observed variations in the IC_50_ values varied from 48.22 µl to 21.75 µl. The control treatment exhibited the highest IC_50_ value on the 9th day (48.22 µl), with no statistically significant difference observed with the treatments of 0.001 mM and 0.01 mM MEL treatments on the same day, as well as the control on the 6th day, The lowest IC50 value was observed in the 0.1 mM treatment on the 3rd day (21.75 µl). The self-protection property of raspberry is attributed to the presence of a diverse phenolic family profile^62^, including anthocyanin and ellagitannin compounds, which contribute significantly to the fruit's strong antioxidant properties. Previous studies^63–66^ demonstrated that approximately 75% of the antioxidant property of raspberry is related to anthocyanin and ellagitannin compounds, with the remaining percentage attributed to vitamin C (20%) and other compounds (5%). The results of the present study represented that MEL plays a significant role in enhancing the antioxidant properties of raspberries by increasing the amount of anthocyanin and, to some extent, AA and maintaining their levels during storage. Considering the inverse relationship between antioxidant activity (DPPH) and the IC_50_ value, the MEL treatment confirmed an increase in antioxidant activity by decreasing the IC_50_ value. These findings underscore the potential of MEL treatment in enhancing the antioxidant properties of raspberry fruit.

### Enzymatic activity (phenylalanine ammonia-lyase, tyrosine ammonia-lyase, and catalase)

The MEL treatment, at varying concentrations, led to a significant increase in the activity of PAL and CAT enzymes, while no significant increase was observed in the activity of the TAL enzyme (*P* ≤ 0.01). The results indicated that different concentrations of the MEL treatment were able to increase the activity of the PAL enzyme at all studied times compared to the control sample (Fig. 5D). The level of PAL enzyme activity varied from 167.07 to 225.9 nm/g FW/min, with the highest level was found in the 0.001 mM treatment on the 3rd day, while the lowest level was observed in the control treatment on the 9th day. In contrast to the PAL enzyme, the TAL enzyme did not show a significant increase in activity in response to MEL treatments. While all different concentrations of MEL led to an increase in the activity of the TAL enzyme compared to control treatments, this increase was not significant, except for the 0.1 mM treatment on the 3rd day. The highest level of TAL enzyme activity was observed in the treatment of 0.1 mM on the third day (9.55 nm/100 g FW), which was statistically significantly different compared with other concentrations of MEL and the control sample, However, no significant difference was observed in other concentrations of MEL compared to the same and the control sample (Fig. 5E).

The CAT enzyme showed the most significant changes, with melatonin treatment at all concentrations significantly increasing its activity. The highest (0.69 u/g FW) and lowest (0.19 u/g FW) levels of the CAT enzyme activity were found in the 0.1 mM treatment on the 3rd day and the control treatment on the 9th day, respectively (Fig. 5F). Anthocyanins and flavonoids are important components of raspberry, which are produced through shikimic acid biosynthesis. Phenylalanine ammonia-lyase and tyrosine ammonia-lyase are key enzymes in this biosynthesis pathway, deaminating phenylalanine or tyrosine to cinnamic or coumaric acid. These enzymes also play a role in enhancing the plant’s resistance system to diseases. Additionally, antioxidant enzymes such as CAT contribute to strengthening the plant’s resistance system. Increasing the activity of these enzymes not only increases the resistance of fruits to various stresses but also improves the nutritional quality and health-promoting properties^67–69^. It is suggested that the MEL treatment induces and increases the expression level of PAL and CAT enzymes and, to some extent, TAL in cold storage. This increased activity increases the level of secondary compounds and antioxidant properties of the fruit. Several reports have documented the increase in activity of various enzymes, including PAL and others involved in the biosynthesis of secondary compounds^41–43,70,71^.

### Hierarchical cluster analysis (HCA) with the heat map and scatter plot

**Figure 6:**
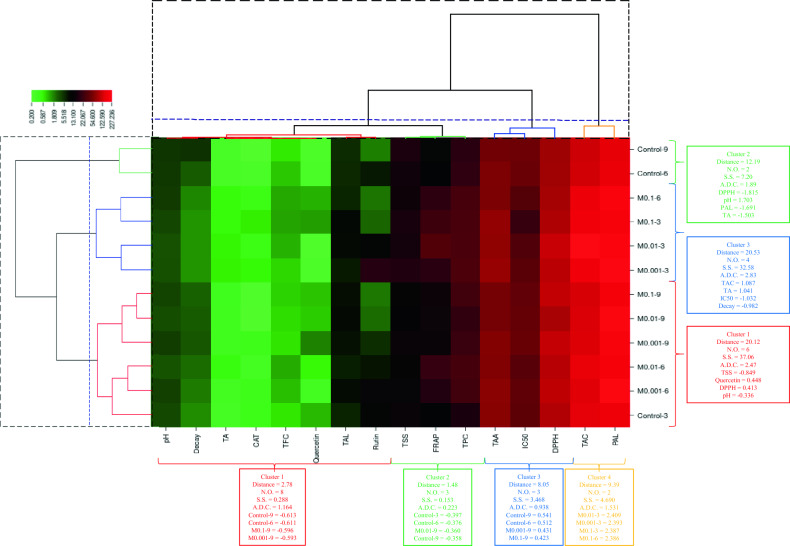
Classification and correlation of treatments and variables studied based on the hierarchical cluster analysis of treatments and variables with Ward’s method.

**Figure 7:**
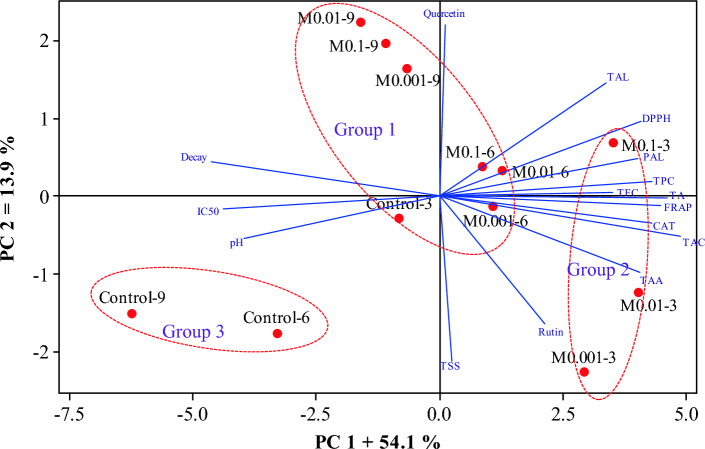
Classification and correlation of treatments studied based on variables’ scatter plot of treatments drawn based on the two first principal components.

As displayed in Fig. 6, the clustering analysis using Ward’s method grouped the 16 studied variables into four groups, with pH, decay, TA, CAT, TFC, quercetin, TAL, and rutin in the first group. Total soluble solids, FRAP, and TPC, as well as TAA, IC_50_, and DPPH variables, were placed in the second and third clusters respectively. Phenylalanine ammonia-lyase and tyrosine ammonia-lyase were in the last group, with the highest amount observed in M0.1–3, M0.1–6, M0.01–3, and M0.001–3 treatments. The treatments were further grouped into three clusters based on the 16 variables (Fig. 6). The first group, consisting of M0.1–9, M0.01–9, M0.001–9, M0.01–6, M0.001–6, and control-3 treatments, is characterized by lower TSS and pH levels and higher quercetin and DPPH values compared to the other groups. The treatments M0.1–6, M0.1–3, M0.01–3, and M0.001–3, placed in the second group, were characterized by high TAC and TA levels and lower IC_50_ and decay. On the other hand, control-6 and control-9 were in the third group, distinguished by higher pH levels and lower DPPH, PAL, and TA values. The principal components analysis confirmed the grouping of the treatments observed in the heat map. The scatter plot with two PC1: 54.1% and PC2: 13.9% showed that the treatments were divided into three groups based on two principal components, with group 1 including M0.1–9, M0.01–9, M0.001–9, M0.1–6, M0.01–6, M0.001–6, and control-3 treatments, and groups 2 and 3 encompassing M0.1–3, M0.01–3, and M0.001–3, as well as control-6 and control-9, respectively (Fig. 7). In general, the grouping with the heat map was almost confirmed using the scatter plot.

### Correlation

**Figure 8:**
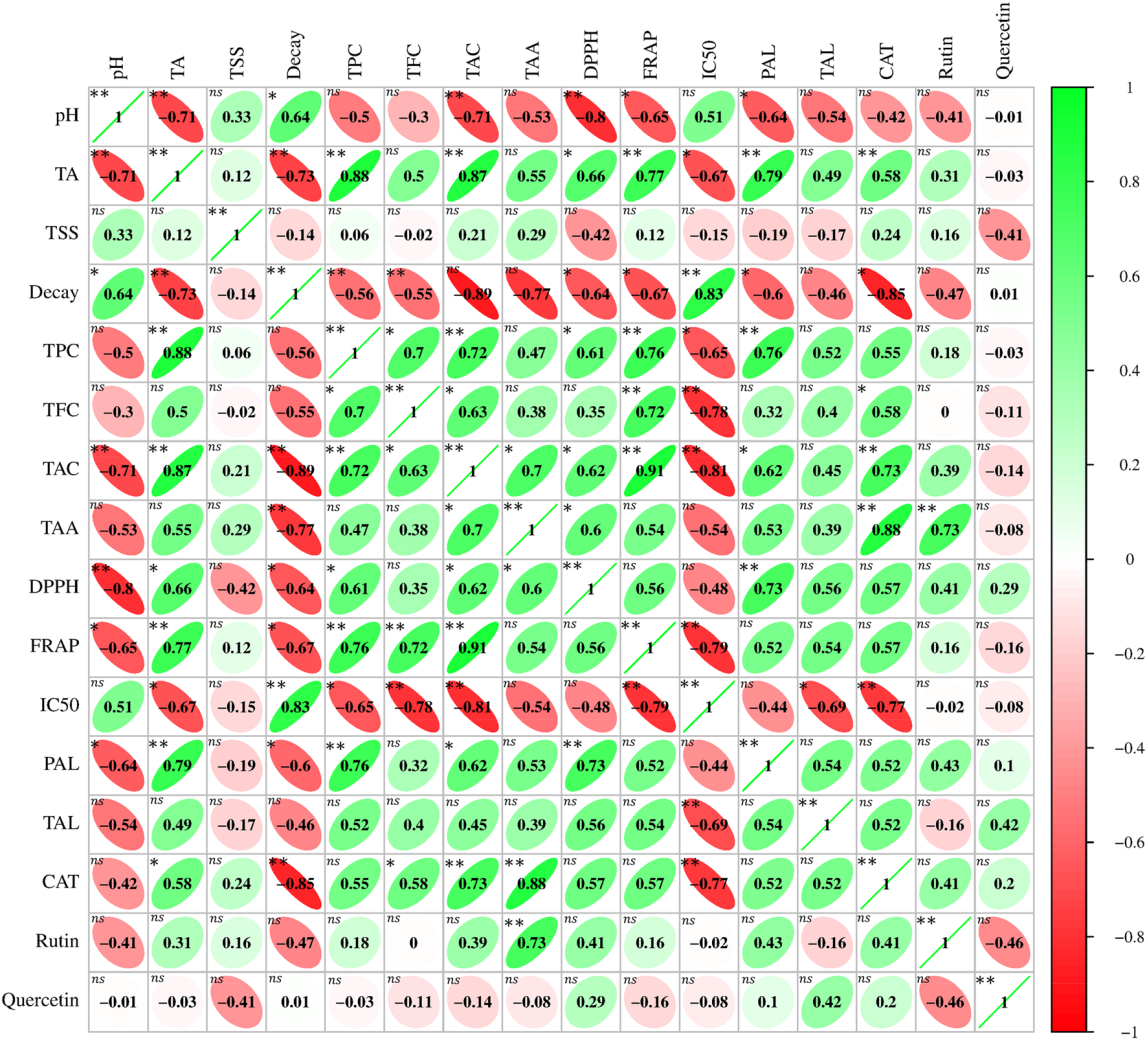
Correlation between phytochemical properties and antioxidant and enzyme activities of treating different concentrations of MEL on raspberry. *MEL* Melatonin.

The relationship between variables helps us better understand their interactions^72^. Pearson correlation was used to show this relationship (Fig. 8). In this correlation, the green and red colors display positive and negative correlations between the two variables, respectively. Based on the results, Pearson coefficient base correlation showed a negative and significant association between TA and pH (-0.71), indicating that the MEL treatment could reduce the rate of decomposition of organic acids into sugars by decreasing the respiration rate^73,74^. Considering that raspberry secondary compounds such as phenols, flavonoids, anthocyanins, and vitamin C have strong antioxidant activities. The existence of a positive and significant correlation between these variables confirms this hypothesis. The results revealed a positive and significant correlation between TPC (+ 0.61), TAC (+ 0.62), and TAA (+ 0.6) with antioxidant activity (DPPH). In addition, there was a positive and significant correlation between TPC (+ 0.76), TFC (+ 0.72), and TAC (+ 0.91) with antioxidant activity (FRAP). In previous studies, a positive correlation has been reported between TPC, TFC, and TAC with the antioxidant activity (DPPH and FRAP) in Damask Rose^72^, raspberry^75^, *Phaleria macrocarpa*^76^, and wild vegetables^77^. Phenylalanine ammonia-lyase and tyrosine ammonia-lyase enzymes play an essential role in the production of raspberry secondary metabolites. The existence of a significant positive correlation between the PAL enzyme and TPC (+ 0.76) and TAC (+ 0.62) proves this issue. Other correlations between traits are shown in Fig. 8.

## Conclusion

The findings of this study demonstrated that the MEL treatment acts as an elicitor, enhancing the activity of key enzymes involved in phenolic biosynthesis, such as PAL, as well as antioxidant enzymes like CAT. This increase in enzyme activities leads to a higher accumulation of phenolic, flavonoid, and anthocyanin compounds, thereby improving antioxidant properties, inhibiting post-harvest decay, and extending post-harvest life. The results indicate that different concentrations of MEL have diverse effects on enzyme and antioxidant activity as well as phytochemical properties, However, the treatment of 0.001 mM MEL had more pronounced effects on the levels of TA (0.619 g/100 ml juice), TAA (60.29 g AA/g FW), rutin (16.72 µl/ml juice), quercetin (1.46 µl/ml juice), antioxidant (FRAP) (30.50 nmol Fe^++^/ml juice), and enzyme (PAL) activities (225.69 nm/g FW/min). Therefore, this concentration is recommended for treating and extending the shelf life of raspberry fruits.

## Data Availability

The datasets used and/or analyzed during the current study are available from the corresponding author upon reasonable request.
